# A New Transalveolar Sinus Lift Procedure for Single Implant Placement: The Ebanist Technique. A Technical Description and Case Series

**DOI:** 10.2174/1874210601711010187

**Published:** 2017-03-31

**Authors:** Rosario Rizzo, Vittorio Checchi, Federico Marsili, Antonio Zani, Serena Incerti-Parenti, Luigi Checchi

**Affiliations:** 1 Private practice, Roncadelle (BS), Italy; 2 Department of Medical Sciences, University of Trieste, Trieste, Italy; 3 Department of Biomedical and Neuromotor Sciences, University of Bologna, Bologna, Italy

**Keywords:** Sinus lift, Bone resorption, Extraoral implant placement, Bone graft, Fresh mineralized frozen homologous bone, Case report, Case series

## Abstract

**Background:**

Nowadays, there are many techniques to compensate bone atrophies of the posterior maxilla in order to obtain an implant-supported rehabilitation.

**Objective:**

This case series describes the Ebanist technique: a sinus lift procedure to be used in case of extremely resorbed bone crests (≤3 mm) allowing simultaneous implant placement.

**Methods:**

With a dedicated cylindrical trephine bur, it is possible to harvest a cylinder of bone from a fresh mineralized frozen homologous bone block graft and to simultaneously create a trapdoor on the recipient site. The trapdoor cortical bone is detached from the sinus membrane and removed. Dental implant is placed into the graft before the grafting procedure since the cylindrical block, once inserted in the recipient area, is not able to oppose sufficient resistance to the torque needed for implant placement.

**Results:**

Second-stage surgery and following prosthetic rehabilitation were performed after 5 months. In all cases, implant stability was manually checked and no pathological symptoms or signs were recovered at any follow-up visit.

**Conclusion:**

This technique can be considered a valid procedure for implant therapy on atrophic posterior upper maxillae, when the residual bone crest is extremely resorbed.

## INTRODUCTION

Nowadays, there are many techniques available to treat bone atrophies of the posterior maxilla in order to obtain a functional and esthetic rehabilitation with an implant-supported restoration [1-4]. The sinus lift technique seems to be the most reliable procedure to compensate qualitative and quantitative bone loss through different surgical techniques, access methods and bone graft materials.

The most commonly used and described sinus lift techniques are the lateral and the transalveolar approach. The lateral sinus lift is distinguished by an osteotomy of the lateral wall of the sinus [5], whereas the transalveolar technique is characterized by a transcrestal access to the sinus from the edentulous alveolar bone [6]. Since the early 90’s, many authors [7-9] proposed modifications of these techniques, always obtaining good results in term of implant survival [1,10]. The major limit of these procedures shows up in case of extremely resorbed bone crests (≤3 mm), since this clinical condition makes implants placement and the following prosthetic rehabilitation extremely difficult.

The goal of this paper is to describe a new procedure: the Ebanist Sinus Lift technique (ESL), so-called due to its similarity with the technicality of Italian cabinet-makers of the 16^th^ century. This technique is particularly useful in case of extremely resorbed bone crests (≤3 mm), because it allows a sinus lift with simultaneous implant placement in one single procedure.

The following case series will describe this new technique.

This case series was conducted in full accordance with ethical principles, including the Declaration of Helsinki, and each participant gave a written consent.

## MATERIALS AND METHODS

### Cases Presentation

#### 1 Case #1

**Fig. (1):**
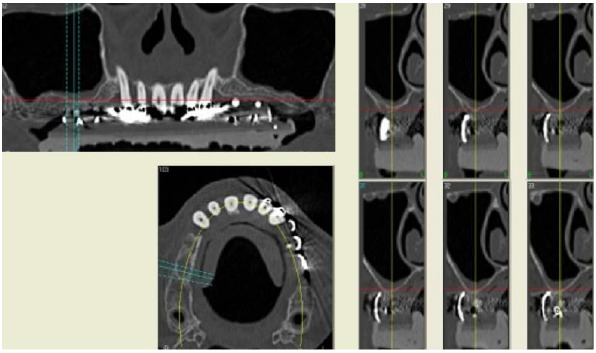
CT scan of the upper maxilla with severe posterior bone atrophy.

In 2007, a 61 years old healthy woman (B.S.) was referred to a private dental practice for rehabilitation of her edentulous upper right posterior sextant, affected by severe bone atrophy (Fig. **1**).

The patient accepted the treatment plan and an implant-supported fixed prosthetic rehabilitation with the ESL technique was performed.

After local anesthesia, a full thickness crestal incision was performed extended from the nearest tooth mesially, to the tuberosity distally. The flaps were then carefully elevated and a buccal horizontal periosteal release incision was performed to mobilize the soft tissues in order to be able to cover completely the graft.

**Fig. (2):**
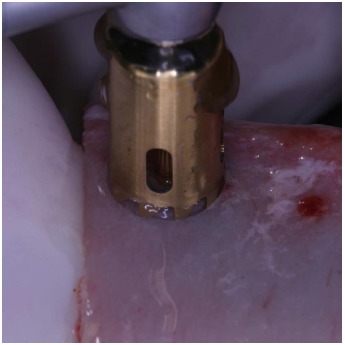
Trephine burs with increasing diameters are used to harvest a cylinder of bone from the fresh bone block and to create a trapdoor on the recipient site with the same diameter. The small hole in the middle of the circle is useful to guarantee the stability of the bur.

A fresh mineralized, non-irradiated, frozen, homologous bone graft was used, provided by the National Bank of Tissue - General Hospital of Treviso (Italy), certified by the National Health Ministry. The ELS technique is simplified by the use of special trephine burs (Physioplant Dental Implant System, Roncadelle, Italy). This system is a set of 4 burs with increasing diameter that allows the surgeon to harvest a cylinder of bone from the fresh block graft and to simultaneously create with the same diameter a trapdoor on the recipient site (Fig. **2**).

**Fig. (3):**
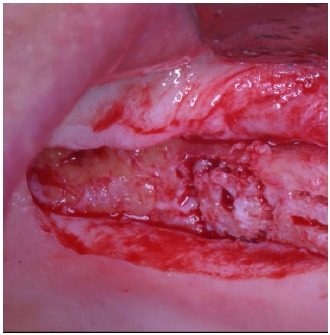
Trephine burs with increasing diameters are used to harvest a cylinder of bone from the fresh bone block and to create a trapdoor on the recipient site with the same diameter. The small hole in the middle of the circle is useful to guarantee the stability of the bur.

**Fig. (4):**
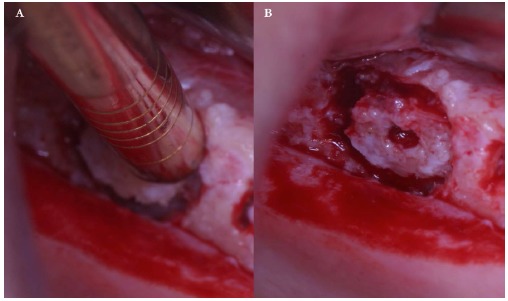
After the creation of the trapdoor, the sinus membrane is detached from the thinned cortical bone that is gently pushed within the sinus.

These trephines have a small cylindrical pivot at the center that takes engagement in a small 2 mm-diameter hole previously performed with a round bur, both on the recipient site and on the graft block. This device does not swing, granting an extremely precise cut and consequently a correct insertion of the graft into the planned recipient area. A trapdoor was created on the patient ridge with the exact diameter of the bone graft previously prepared (Fig. **3**) and the host cortical bone was detached from the sinus membrane and gently pushed within the sinus (Fig. **4a**, **b**).

The Schneiderian membrane was raised with caution, for only few millimeters (5-6 mm) around the trapdoor, enough to expose bony anchorage areas and to set in the graft without any tensions.

**Fig. (5):**
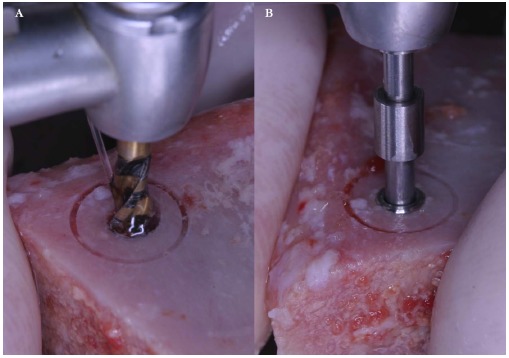
Implant placement into the graft before grafting procedure.

The implant (Physioplant Dental Implants, Roncadelle, Italy) was placed into the graft before the grafting procedure (Fig. **5a**, **b**) because the cylindrical block, once inserted in the recipient area, could not be able to oppose sufficient resistance to the torque needed to place the implant.

**Fig. (6):**
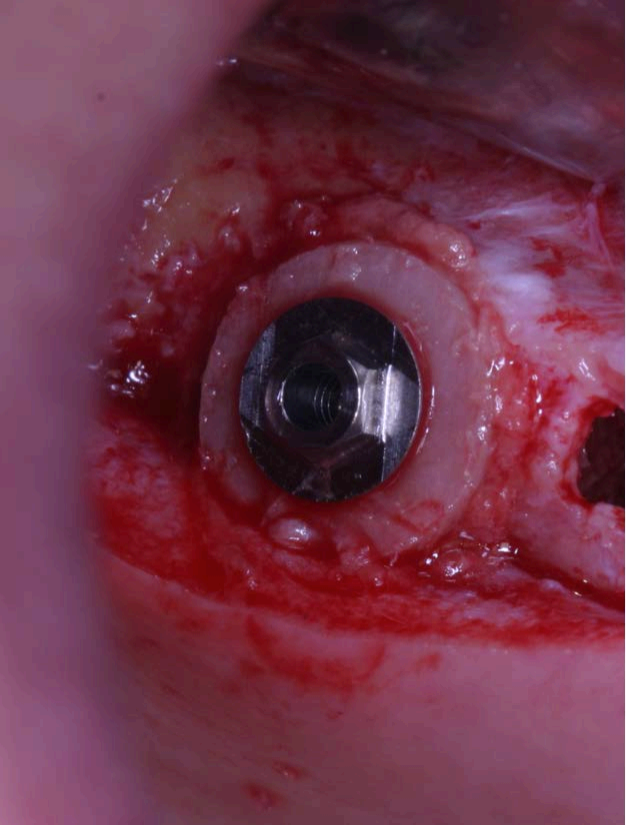
Perfect match obtained between the graft and the recipient site, in order to achieve a good stability. (The second implant placed mesially has been placed in an experimental way, not described in this article).

Consequently, the shape of the cylindrical graft matched perfectly the size of the recipient site, simplifying the insertion of the allograft. After its placement, the cortical part of the block was gently thinned down until its removal, in order to allow its complete substitution during the healing processes (Fig. **6**). A slow resorbable membrane was used to completely cover and protect the graft, then the flaps closure was obtained with horizontal mattress sutures.

#### 2 Case #2

**Fig. (7):**
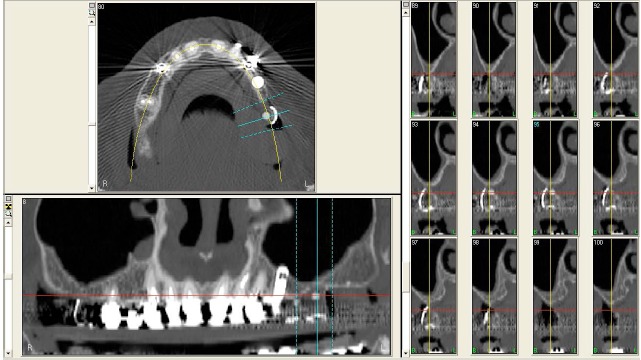
CT scan of the upper maxilla with severe posterior bone atrophy.

An implant-supported fixed prosthetic rehabilitation after sinus lift performed with the ESL technique was suggested to a 66 years old healthy woman (A.A.) with monolateral posterior edentulism of the upper left maxilla (Fig. **7**).

**Fig. (8):**
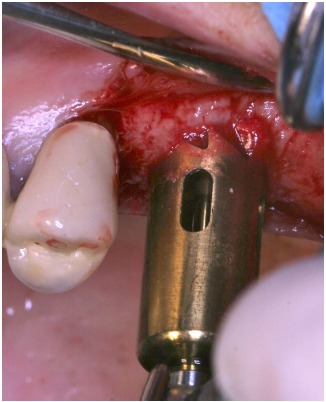
Creation of the trapdoor with the ESL dedicate bur.

**Fig. (9):**
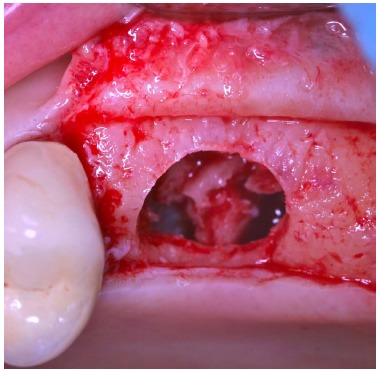
After the creation of the trapdoor, the sinus membrane is detached from the thinned cortical bone that is removed.

After local anesthesia, a full thickness crestal incision was performed on the upper right posterior edentulous ridge. Using the ESL dedicated burs, a circular trapdoor was created in the posterior area (Fig. **8**) and the host cortical bone was detached from the sinus membrane and removed (Fig. **9**).

**Fig. (10):**
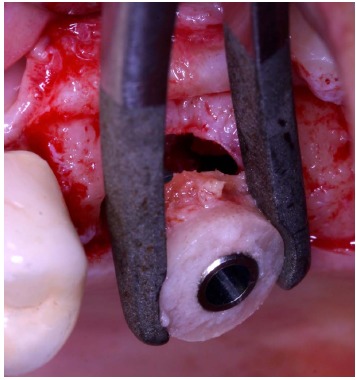
Perfect match obtained between the graft and the recipient site.

A cylindrical bony graft was harvested from a block of fresh mineralized, non-irradiated, frozen, homologous bone, using the same diameter ESL bur used to create the trapdoor. A dental implant (Physioplant Dental Implants, Roncadelle, Italy) was placed into the graft before its placement in the recipient site. Since the shape of the cylindrical graft matched perfectly the size of the recipient site, the combination of graft and implant was inserted into the recipient site (Fig. **10**).

A slow resorbable membrane was used to completely cover the graft and primary closure was obtained with horizontal mattress sutures.

#### 3 Case #3

**Fig. (11):**
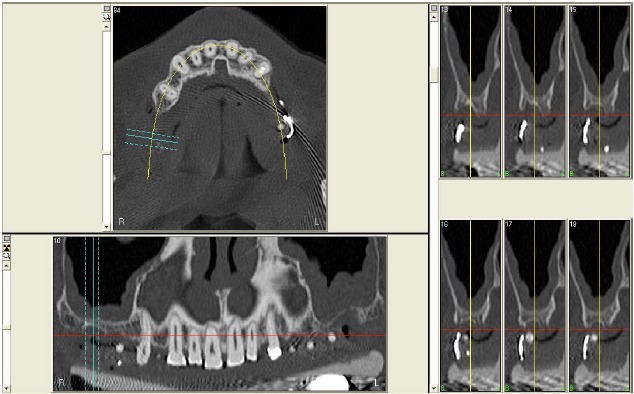
CT scan of the upper maxilla with severe posterior bone atrophy.

A 67 years old healthy man (R.A.) was referred in a private dental practice asking for a fixed rehabilitation. The patient presented a bilateral edentulous posterior atrophic bone crest of the upper maxilla (Fig. **11**). The left side was treated with traditional implant therapy in the premolar area, and with implant placement in association with the ESL technique in the molar area.

After local anesthesia and full thickness crestal incision, a circular trapdoor was performed in the molar area, using an ESL dedicated bur with adequate diameter.

The host cortical bone was detached from the sinus membrane and removed. The graft chosen for this case was a block of fresh mineralized, non-irradiated, frozen, homologous bone. From this graft, selecting the same dedicated ESL bur used to create the trapdoor, a cylindrical block was created and a dental implant (Physioplant Dental Implants, Roncadelle, Italy) was placed in it.

**Fig. (12):**
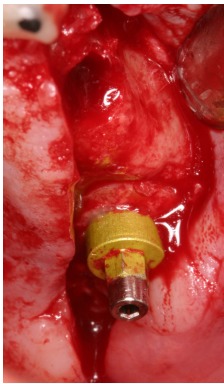
Simultaneous placement of the graft and the implant in the recipient site.

Taking advantage of the perfect match between graft and recipient site, the cylindrical block with the dental implant was placed and resulted clinically stable (Fig. **12**). A slow resorbable membrane was used to cover the graft. Flaps were finally sutured with horizontal mattress sutures.

#### 4 Case #4

**Fig. (13):**
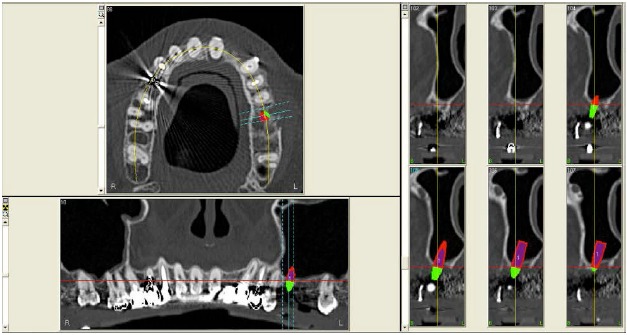
CT scan of the upper maxilla with severe posterior bone atrophy.

A 54 years old healthy woman (M.) asked for an implant-supported fixed rehabilitation of her upper posterior left sextant. Due to the extremely atrophic crest (Fig. **13**), it was chosen to perform implant therapy in association with the ESL technique.

After local anesthesia and full thickness flap raising, using a dedicated circular bur, a trapdoor was created on the first molar area.

**Fig. (14):**
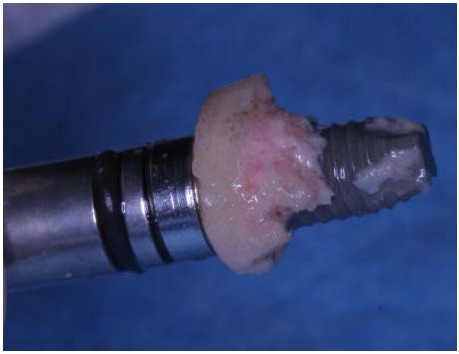
Implant placement into the cylindrical graft before grafting procedure.

**Fig. (15):**
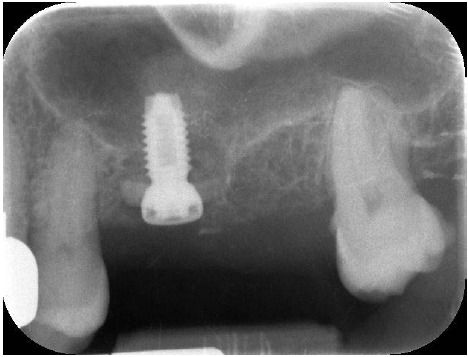
Periapical x-ray showing the implant and the graft in place after sutures.

Cortical bone was removed from the sinus membrane and a block of fresh mineralized, non-irradiated, frozen, homologous bone was chosen for this case. From the graft, a cylindrical block was created, using a bur 1 mm bigger in diameter than the one used to crete the trapdoor. A dental implant (Physioplant Dental Implants, Roncadelle, Italy) was placed in the graft (Fig. **14**) before the grafting procedure. The graft perfectly matched the recipient site and appeared clinically stable (Fig. **15**). A slow resorbable membrane covered the graft and flaps were sutured.

## RESULTS

Post surgical instructions were the same for all cases. A 2 g dose of amoxicillin with clavulanic acid was administered preoperatively, followed by 1 g twice daily for 6 days. Ibuprofen (600 mg) was recommended immediately after surgery together with 4 mg of betamethasone. A cold/soft diet and appropriate oral hygiene were recommended for 2 weeks. Patients were not allowed to wear removable dentures before implant uncovering. Patients were seen after 7 days for sutures removal and after other 14 days for wound healing control.

In all cases, postoperative recovery was uneventful: soft tissues healed well and no signs of inflammation were present.

Dental implants remained covered underneath the soft tissues and monthly follow-ups were planned for 5 months, when the second surgery was scheduled to uncover the implants.

For each implant, stability was manually checked tightening the abutment screws with a 20 Ncm torque. No pathological symptoms or signs (implant mobility, peri-implant probing depth or peri-implant bleeding on probing) were recovered at any follow-up visit (provisional and definitive prosthetic loading time) for each implant.

**Fig. (16):**
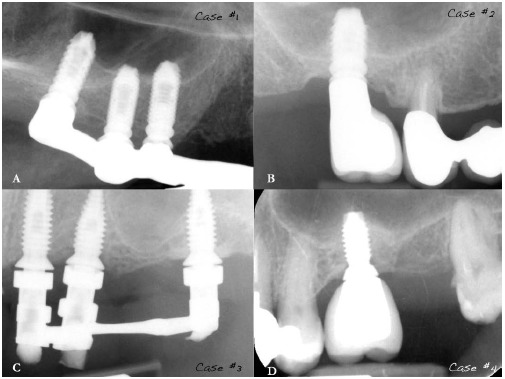
Periapical x-rays of cases #. 1 **(a),** #. 2 **(b),** #. 3 **(c)** and #. 4 **(d)** showing no pathological signs respectively on implant #. 1.6 after 6 years from the grafting procedure, on implant #. 1.6 after 3 years, on implant #. 2.6 after 4 years, and on implant #. 2.6 after 3 years. The gap visible between implants and abutments is due to the presence of a radiolucent shock adsorber considered by the used implant system.

Radiographic assessments of case #. 1 showed no pathological signs 6 years (Fig. **16A**) after implant placement and no peri-implant bone loss.

Cases #. 2, #. 3 and #. 4 also didn’t show pathological signs nor bone loss respectively 3 years (Fig. **16B**), 4 years (Fig. **16C**) and 3 years (Fig. **16D**) after implant therapy.

## DISCUSSION

This case series shows that the association between the Ebanist technique and the use of fresh frozen cancellous bone graft seems to be able to obtain remarkable outcomes. This technique is rather simple and requires only one surgical procedure, since it allows the placement of graft and implants at the same time. The ESL can be considered a valid procedure for simultaneous implant therapy and bone augmentation on atrophic posterior upper maxillae, when the residual bone crest is extremely resorbed (≤3 mm).

Grafting the floor of the maxillary sinus has become the most common surgical intervention to increase insufficient alveolar bone height before the placement of dental implants in the posterior maxilla [1].

The indication for a sinus lift procedure is clear when the residual bone shows 10 mm or less height in the posterior maxilla [11]. The use of this lateral approach is able to increase the vertical bone height more than 9 mm [12].

On the contrary, the use of the osteotome technique is able to gain about 3 to 9 mm of vertical bone height increase [13].

The selection of the suitable sinus lift technique is therefore mainly founded on the height of pre-implant residual bone. When the residual bone is more than 5 mm of height, the transcrestal technique is the most indicated [2]. On the other hand, when the residual bone height is 5 mm or less, the lateral window approach is indicated [14].

Limitations for implant placement in the posterior maxilla are related for sure to residual bone height and width but also to the property of maxillary bone. Poor bone quality and quantity in posterior maxilla are considered the reasons for the decreased primary implant stability and, therefore, the increased failure rates [15].

It has been shown that the implant survival rate decreases with the redurction of the pre-implant residual bone height [16]. Most of this decrease in survival rate is expected when the pre-implant bone height is less than 5 mm [17]. This observed correlation between pre-implant residual bone height and survival rate reveals an important clinical point that requires further consideration in developing implant treatment plans in atrophic posterior maxillae.

The authors suggest the use of a fresh frozen cancellous bone graft (FFB) for the ESL technique. Due to its handling process, FFB undergoes a procedure of removal of the antigenic sites from the bone surface, showing angiogenesis induction and enhanced immune tolerance [18]. Moreover, in case of fresh bone harvested from young donors, most of the bone morphogenetic proteins (BMP) are well conserved and represented [19, 20].

Concerning the clinical outcome of prosthetic rehabilitation, it has been shown [21] that implants inserted simultaneously with FFB grafts (but not immediately loaded) have similar survival and success rates to those placed in no grafted sites or in areas grafted with autogenous bone [22].

Different studies [21, 23] showed that FFB is a reliable graft material for the reconstruction of lost bony ridge volumes with simultaneous implant placement.

Concerning the recipient site, great importance is given to the bucco-palatal bone width in its most coronal portion and to the presence of possible anchorage anatomical areas. For example, the presence of a bone septum or even the convergence of palatal and/or buccal bony walls of the sinus floor may be strategic areas useful to improve graft stability. The minimum ridge width required to perform the surgery with the cylindrical graft is 7 mm, because a graft with smaller dimensions could fracture during implant insertion.

Contrary, the thickness of the residual bone in the recipient area is quite irrelevant. The reason is that both the trapdoor and the graft are designed with an equal width and diameter to that measured between the palatal and the buccal bony walls of the sinus.

In order to perform a more precise surgery it is advisable to create a surgical template that allows the correct positioning of the implants, facilitates the development of anchorage areas and permits a precise placement of the graft. This same template will also help to easily locate the implants during the second stage surgery.

## CONCLUSION

All the modifications to the original sinus lift techniques presented in the literature [1, 7-10, 24] have shown good results in terms of bone reconstruction, vertical bone increase, graft compatibility, implant success and implant survival. However, when the treatment plan considers a sinus lift with simultaneous implant placement, none of these techniques is useful in case of extremely resorbed bone crests with 3 or less mm of residual bone.

Following the positive results achieved in this case, it would be useful to prepare a randomized clinical trial to compare the ELS technique with other transalveolar sinus lift procedures.

## LIST OF ABBREVIATIONS

BMP: = Bone Morphogenetic Proteins
ESL: = Ebanist Sinus Lift Technique
FFB: = Fresh Frozen Cancellous Bone Graft
g: = gram(s)
mg: = milligrams
mm: = millimeters
